# Intelligence

**Published:** 1808-05

**Authors:** 


### INTELLIGENCE.

Experiments lately made at Venice, shew, that the oil of the Chinese radish is preferable to any other kind known, not only for culinary purposes, and giving light, but also as a medicine. From the experiments lately made by Dr. Oliviero, it is found to be extremely useful in rheumatic and pulmonary affections, and has been employed with much success in convulsive coughs. It is not liable to spoil by keeping, like other oils, nor is the plant injured by the strongest frosts. In May and June the seed is gathered, which is very abundant.

The light of the Pyrosoma Atlanticum has not been described by naturalists. M. Peron, in his late voyage, observed this animal in between the 3d and 4th degrees of N. latitude. Its luminous property renders it one of the most splendid of all known zoophites. The darkness was intense when it was first discovered, the wind blew with violence, and the progress of the vessel was rapid. All at once there appeared, at some distance, a vast sheet of phosphorus floating upon the waves before the vessel. The ship having passed through this brilliant part, the crew discovered that the light was occasioned by an immense number of small animals, which swam at different depths, and assumed various forms. Those which were deepest looked like red-hot shot, and those on the surface resembled tubes of red-hot iron. Some were soon caught, and they were found to vary in size from three to seven inches. All the exterior surface was bristled with thick oblong tubercles, shining like so many diamonds, and these seemed to be the principal seat of phosphorescence. In the inside there appeared a multitude of oblong narrow glands, which possessed the phosphoric property in a high degree. The colour, when at rest, is an opal yellow mixed with green; but on the slightest motion, or spontaneous contraction, the animal instantly becomes luminous. As it loses its phosphorescence it passes successsively through a number of tints, such as red, orange, green, and azure blue.

The following directions are given in the foreign Journals for preparing a composition that will resist the action of fire and water.—Take half a pint of milk, and mix with it an equal quantity of vinegar, so as to coagulate the milk. Separate the curds from the whey, and mix the latter with the whites of four or five eggs, after having beat them well up. The mixture of these two substances being complete, add quick lime to them which has passed through a sieve, and make the whole into a thick paste of the consistency of putty. If this mastic is carefully applied to broken bodies, or to fissures of any kind, and properly dried afterwards, it resists water and fire.

Dr. Clutterbuck, Member of the Royal College of Physicians, and one of the Physicians to the General Dispensary, Aldersgate Street, will begin his Summer Course of Lectures on the

Theory and Practice of Physic, and the Materia Medica, on Monday the 30th of May, at eight o'clock in the morning precisely. Further Particulars, with Terms, and a Prospectus, may be had on application at the Dispensary, or at No. 17, St. Paul's Church Yard.

Dr. Clough, Physician, Manmidwife to the St. Mary-le-bone General Dispensary, &c. will, on Monday the 23d of May, at ten o'clock in the morning, commence his Course of Lectures on the Theory and Practice of Midwifery, including the Diseases of Women and Children, and their Treatment. A Syllabus and Prospectus of the Course may be had of Dr. Clough, No. 68, Berner's Street, Oxford Street; J. Callow, Crown Court, Soho; and at the Dispensary, 77, Welbeck Street, Cavendish Square. An Evening Course, for the convenience of such Students as cannot attend the Morning Lectures, will be given on Tuesdays, Thursdays, and Saturdays, at seven. The first Evening Lecture will be on Tuesday the 24th instant.

Mr. John Taunton's Summer Course of Lectures on Anatomy, Physiology, Pathology, and Surgery, will commence on Saturday, May 28, 1808, at eight o'clock in the evening. Further particulars may be learned on application to Mr. Taunton, Greville Street, Hatton Garden.

Mr. Parkinson's Second Volume of Organic Remains of a Former World, will be published in the beginning of June. It will contain twenty plates, representing nearly two hundred different Fossils of Zoophyte Remains, coloured after Nature, among which are the mineralized remains of upwards of twenty species of the Encrinus; the greater number of which have been found in this island.

Mr. John Ayrton Paris, of Caius College, Cambridge, and Fellow of the Royal Medical Society, Edinburgh, has now in the press a Compendium of Modern Chemistry, in the Latin language; a small work intended as the *fidus Achates* of the medical as well as chemical philosopher. It treats not only of the principal subjects in chemistry, but unfolds the processes by which the different compounds of the London and Edinburgh Pharmacopœia are prepared, and the theories upon which such operations are founded. The analyses also of animal and vegetable bodies are as fully given as the prescribed limits of a compendium will allow. This work will also afford easy instructions for the disciple of the Stahlean School to become the proselyte of Lavoisier. The language in which it is written, will, it is hoped, render it no inconsiderable assistant to those desirous of writing or speaking medical Latin. And if it be remembered that no such publication has appeared in the chemical department subsequent to Boerhaave, the hope may not be presumptuous.

